# Quantum dot therapeutics: a new class of radical therapies

**DOI:** 10.1186/s13036-019-0173-4

**Published:** 2019-05-29

**Authors:** Max Levy, Partha P. Chowdhury, Prashant Nagpal

**Affiliations:** 1 Chemical and Biological Engineering, University of Colorado Boulder, Boulder, CO 80303 USA; 2 Renewable and Sustainable Energy Institute, University of Colorado Boulder, Boulder, CO 80303 USA; 3 Materials Science and Engineering, University of Colorado Boulder, Boulder, CO 80303 USA

**Keywords:** Radical antimicrobials, Multidrug-resistant superbugs, Quantum dot therapeutic, Reactive oxygen species

## Abstract

Traditional therapeutics and vaccines represent the bedrock of modern medicine, where isolated biochemical molecules or designed proteins have led to success in treating and preventing diseases. However, several adaptive pathogens, such as multidrug-resistant (MDR) superbugs, and rapidly evolving diseases, such as cancer, can evade such molecules very effectively. This poses an important problem since the rapid emergence of multidrug-resistance among microbes is one of the most pressing public health crises of our time—one that could claim more than 10 million lives and 100 trillion dollars annually by 2050. Several non-traditional antibiotics are now being developed that can survive in the face of adaptive drug resistance. One such versatile strategy is redox perturbation using quantum dot (QD) therapeutics. While redox molecules are nominally used by cells for intracellular signaling and other functions, specific generation of such species exogenously, using an electromagnetic stimulus (light, sound, magnetic field), can specifically kill the cells most vulnerable to such species. For example, recently QD therapeutics have shown tremendous promise by specifically generating superoxide intracellularly (using light as a trigger) to selectively eliminate a wide range of MDR pathogens. While the efficacy of such QD therapeutics was shown using in vitro studies, several apparent contradictions exist regarding QD safety and potential for clinical applications. In this review, we outline the design rules for creating specific QD therapies for redox perturbation; summarize the parameters for choosing appropriate materials, size, and capping ligands to ensure their facile clearance; and highlight a potential path forward towards developing this new class of radical QD therapeutics.

## Introduction

Reduction and oxidation reactions form the core of most significant processes in biology, where the majority of biological interactions, signaling, and basic cellular biology involves either a gain or loss of electrons or ionic species/radicals [1]. Most prominently, many redox species are regulatory and believed to be used for molecular signaling and as activators of stress response [2–6]. Others, however, can cause indiscriminate oxidative damage and dysfunction [7–12]. Chemical reactions such as Fenton chemistry [13], enzymatic conversions, and disproportionation [14], can convert these species into others–such as the conversion of superoxide into hydrogen peroxide, hydroxyl radicals, and peroxynitrite ions [13, 15, 16]. Therefore, careful choice of desired biological targets, mechanistic insights into redox species and their outcome inside a cell, and precise control over their intracellular generation can provide a vital tool for precision or specific killing of cellular species vulnerable to a chosen redox perturbation, that can be triggered by stimuli to act as a therapeutic.

## Designing for a “radical” approach

While many traditional antibiotics have suffered failure against adaptive resistance, a versatile approach to address this dynamic problem is emerging. Where traditional small-molecule antimicrobials struggled with transport into gram-negative pathogen cell walls, nanoparticle-based therapeutics have shown remarkable stability, ease of delivery, and facile transport through cell walls due to their small size [17–21]. Once inside the cell, the nanoparticle or QD therapy can make use of the presence of oxygen, water, and if required, an external trigger. Therefore a wide range of reactive oxygen species (ROS, e.g. superoxide O_2_^●-^, hydroxyl OH^●^, singlet oxygen ^1^O_2_, and hydrogen peroxide H_2_O_2_) and reactive nitrogen species (RNS, e.g. nitric oxide NO^●^, peroxynitrite ONOO^−^) can be formed intracellularly using redox chemistry. Since these species are responsible for a broad range of physiology and pathology in living organisms [22, 23], they have been investigated for such potential applications as cancer therapies and novel antimicrobials. Therefore, specific intracellular generation of these species can drastically affect the specificity of ROS/RNS therapy using the proposed redox perturbation.

**Fig. 1:**
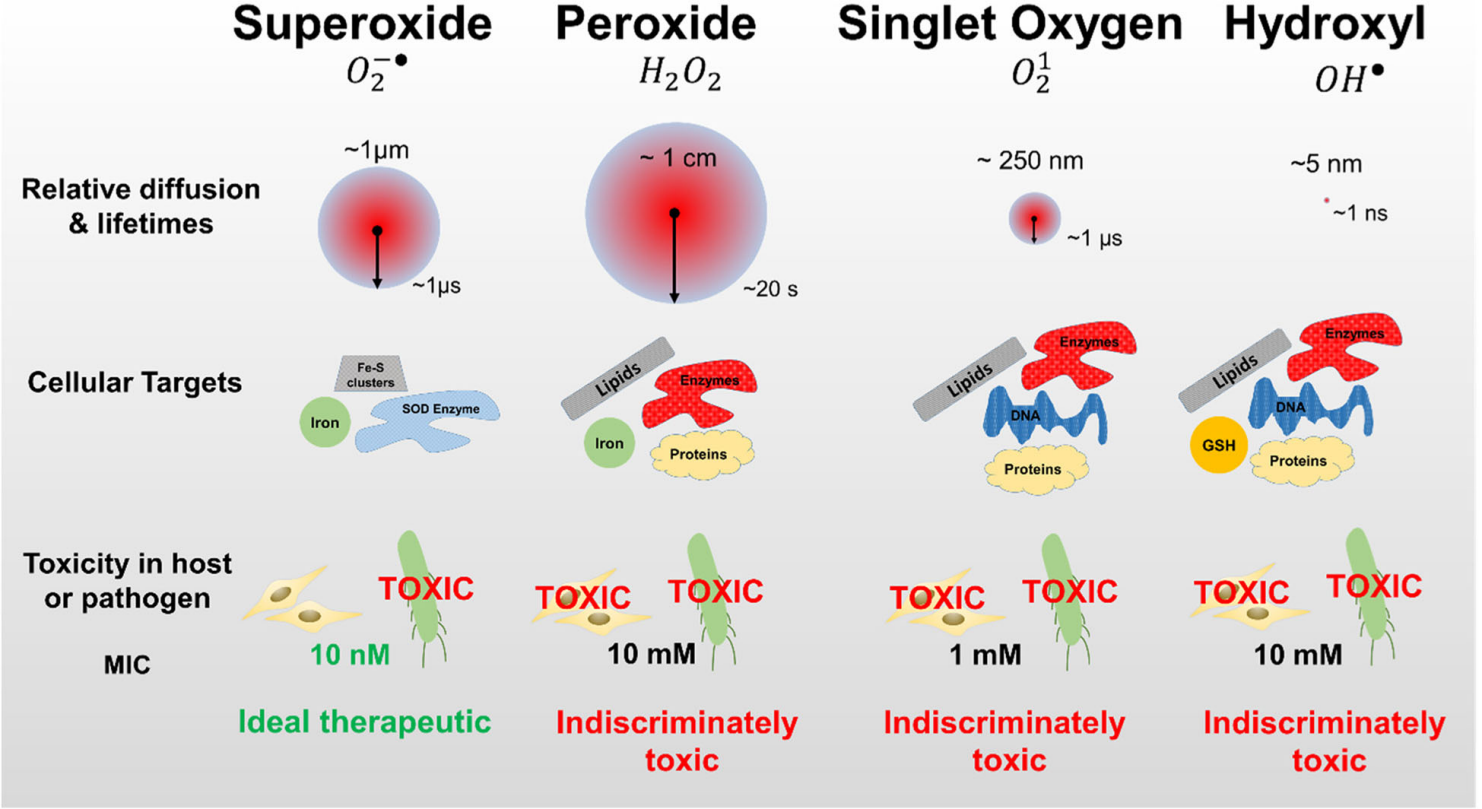
Identifying pathogen vulnerability using redox perturbation with different ROS. Compared to singlet oxygen and hydroxyl radicals, superoxide and peroxide have much longer diffusion lengths and half-lives in the cellular environment (red circles, not to scale) [24, 82]. Singlet oxygen and hydroxyl radicals are also more nonselective – they react rapidly with an abundance of endogenous biomolecules. Superoxide is more selective, partly due to its negative charge, and it reacts with very particular consequential cellular targets such as iron-sulfur clusters [25]. The endogenous bacterial defense against superoxide is less abundant than the defense against nonselective ROS. This leads to a drastically lower observed toxicity threshold when compared to other species [24]. Unlike other ROS, superoxide offers a large window of dosage that yields toxicity in pathogens and nontoxicity in hosts [19, 24]

Recently, our group assessed these different ROS and RNS species as potential therapeutics [24]. Using these species intracellularly, we determined their respective minimum inhibitory concentration (MIC) values. We found a bactericidal effect for several species at high threshold concentrations (singlet oxygen: 1 mM; peroxide: 10 mM; hydroxyl radical > 10 mM; nitric oxide > 1 mM, Fig. 1) [24, 25], where these redox species would be toxic even for host mammalian cells [26–28]. However, superoxide was found to be a potent bactericidal at low nanomolar doses—killing a range of multidrug-resistant (MDR) pathogens without affecting the viability or growth of host mammalian cells in in vitro measurements [19, 20, 24, 29]. This difference in nanotherapeutic toxicity between host and the targeted pathogen is important for designing the safest possible treatment. Biological specificity enables a treatment to effectively clear infections while preserving the host cells. Although the superoxide anion has a high thermodynamic capacity to be a strong oxidant, its lack of reactivity with cellular components at physiological pH (largely due to electrostatic repulsion with negatively charged biomolecules)--except for the inactivation of biosynthetic enzymes containing labile iron-sulfur clusters--is key to its selectivity [25, 30]. Further, the role of iron sequestration in host colonization makes pathogenic bacteria particularly vulnerable to superoxide compared to hosts [31, 32]. Therefore, while several ROS species like hydroxyl radicals are indiscriminate oxidants and can readily oxidize proteins, lipids, and nucleic acids [8, 33], prior studies and our experiments indicate specificity in the of superoxide anions [19, 20, 24, 29, 34, 35]. Given the specificity of superoxide’s mechanism of action, the low MIC value for pathogens and higher tolerance in host mammalian cells, its long lifetime and large diffusion length make it an ideal candidate for selective redox therapy.

### Selective redox activation using quantum states

**Fig. 2:**
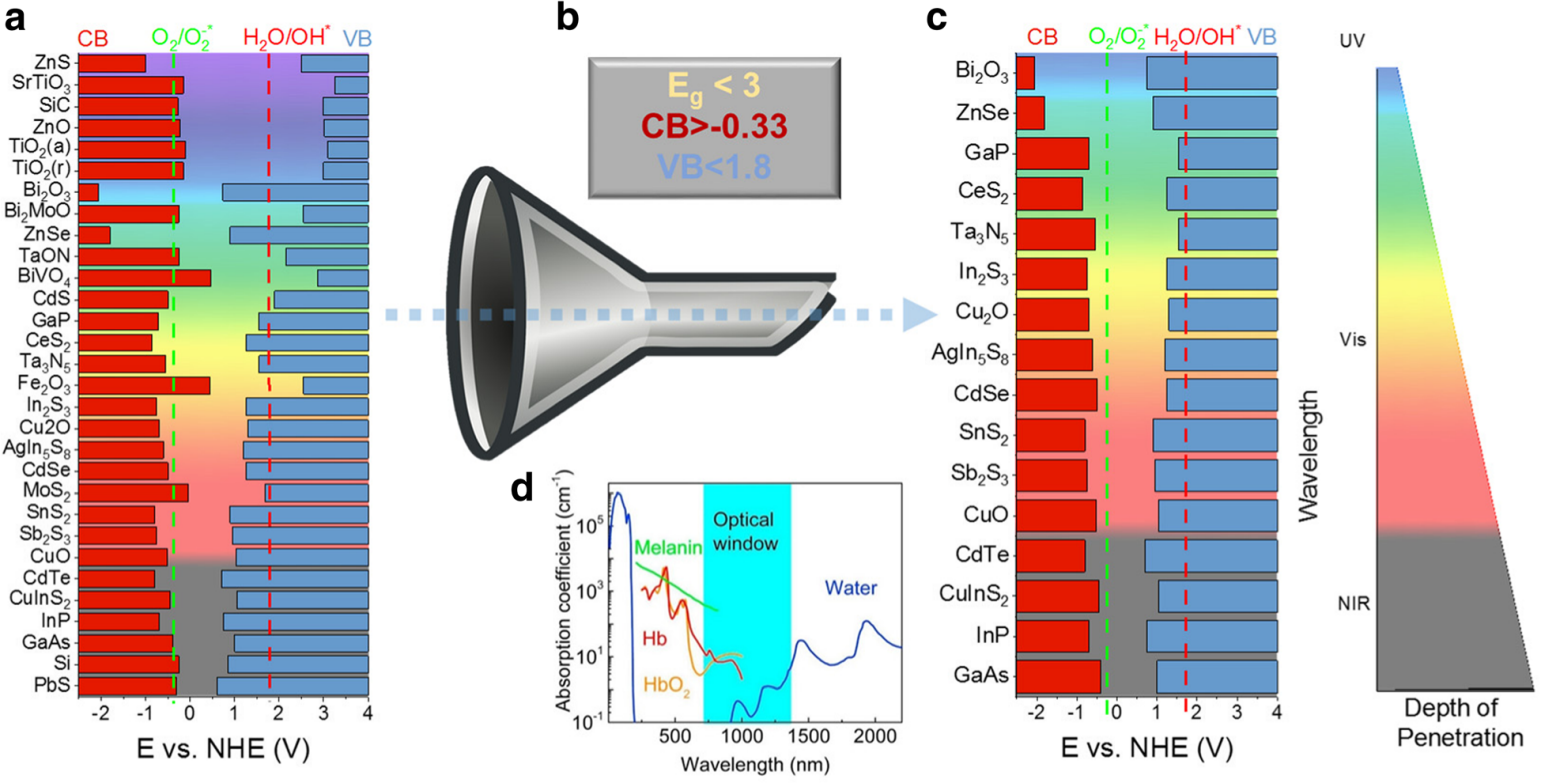
Criteria for material selection for QD therapeutic against MDR superbugs. **a)** Conduction band (red) and valence band (blue) positions for select semiconductors in bulk, according to references cited in reviews herein [83, 84]. Band edge positions shown in relation to thresholds for superoxide (green) and hydroxyl radical (red) generation. Many of these materials only absorb ultraviolet light or are unable to generate superoxide. **b)** By applying rational constraints to this list of materials, we can narrow this (non-exhaustive) list of candidates. **c)** These candidates could potentially generate therapeutic superoxide using visible or NIR light, which penetrates deeper through tissue than UV (**d**), reproduced with permission from the *American Chemical Society*^37^

QDs, or semiconductor nanocrystals, have size-, shape-, and composition-tunable quantum states for reduction and oxidation reactions. These states can be triggered by external electromagnetic radiation like light, and have demonstrated a promising role in non-traditional redox therapy [17–20, 24, 29]. Precise control over their photogenerated electron and hole states provides a unique ability to tailor their photochemistry in the cellular environment, thereby providing control over intracellular redox species. As the first step towards designing an effective QD therapeutic, we assessed a range of different materials, along with their corresponding (bulk) reduction and oxidation states (Fig. 2**a**). To select for specific intracellular generation of superoxide, the reduction potential should exceed − 0.33 V on normal hydrogen electrode (NHE) scale. Simultaneously, to avoid the formation of other non-specific ROS species that can cause indiscriminate cell damage, the oxidation potential should be less than 1.8 V NHE. Using this metric as a selection criterion for selective redox antimicrobial therapy, and classifying the materials on the basis of their nominal (bulk) bandgap values, we obtained a list of ‘favorable’ materials for the proposed QD nanotherapy. Keeping in mind the extinction of light as it enters the skin considering the most common constituents as water, hemoglobin, melanin, etc. [20, 36, 37], there is a window of nominal biological transparency (~ 800–1300 nm wavelength), which narrows the material and bandgap considerations further (Fig. 2b-d). Materials which absorb violet and UV light are less suitable for QD nanotherapy—such short wavelengths of light will be quickly scattered or absorbed near the surface of animal tissue. This lack of penetration would make it extremely challenging to use wide-bandgap materials to treat systemic infections. Red and near-infrared absorbing QDs would be far less susceptible to this issue. Therefore, near-infrared materials like cadmium telluride (CdTe) [19, 20, 24, 29], copper indium sulfide (CIS_2_) [19], indium phosphide (InP), and gallium arsenide (GaAs) could serve as good candidates for selective antimicrobial, material stability, cytotoxicity, and surfaces [17, 18]. Using dopants and bandgap engineering, it is also possible to improve the suitability of some other materials. For instance, carbon QDs and silicon QDs have many reported biological applications in bio-imaging, cancer therapy, as well as some reports describing ROS-mediated therapy [38–41]. Depending on particle size and dopants,the optical properties of these materials can be specifically tailored to decrease the energy of light required for photoactivation [42]. The same approach can also be extended to metal oxide materials.

**Fig. 3:**
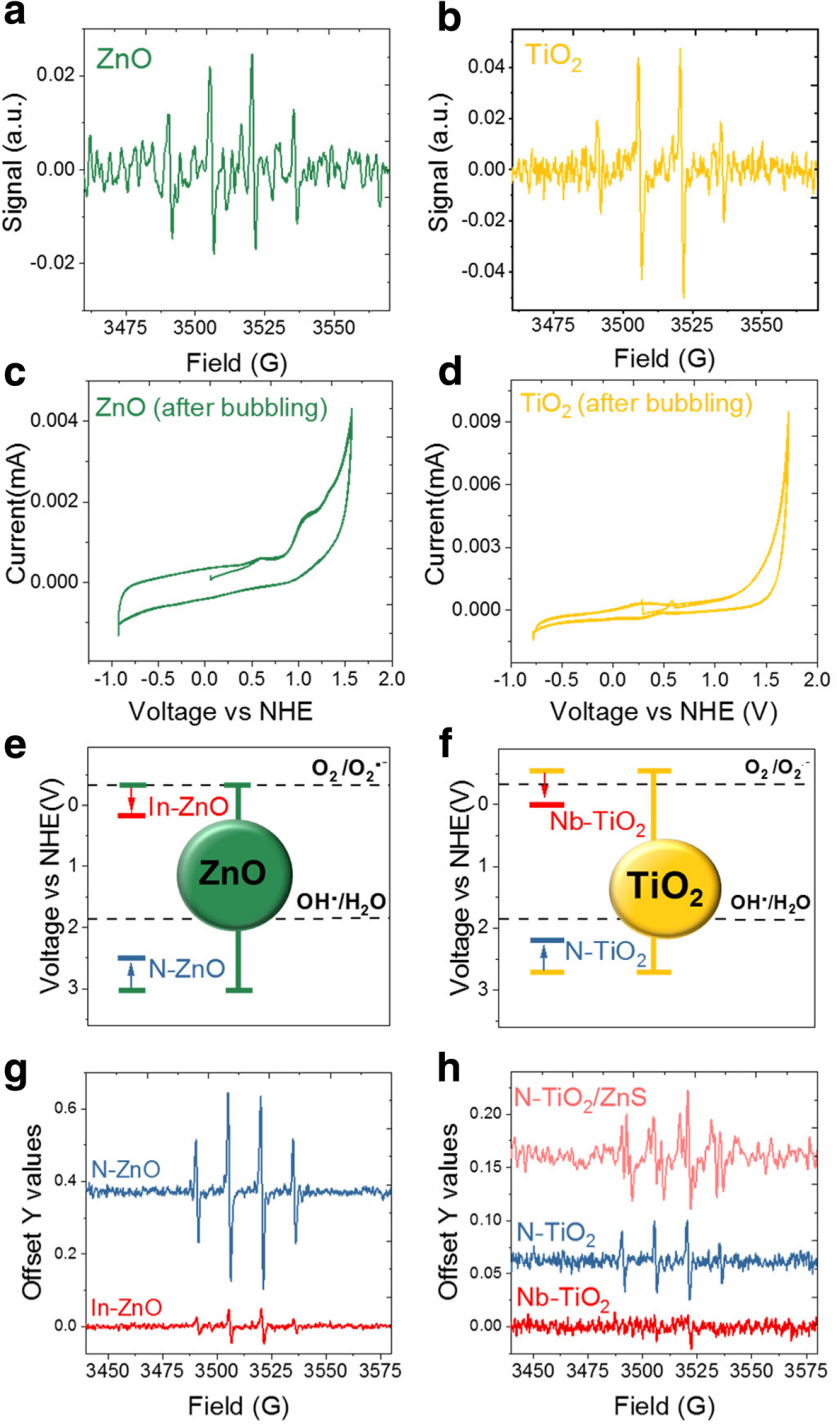
Bandgap and redox state engineering for therapeutic radicals. **a-b)** EPR spectra for UV illuminated ZnO and TiO_2_ nanoparticles_,_ respectively, showing DMPO-OH peaks corresponding to the spin-trapped adduct of hydroxyl radicals. **c-d)** cyclic voltammograms for ZnO and TiO_2_, respectively, in deoxygenated water. Without a source of oxygen, TiO_2_ generates no radical signal but ZnO shows a peak corresponding to hydroxyl radicals – indicating superoxide-generating ability from TiO_2_ but not ZnO. **e-f)** reduction and oxidation state positions for ZnO and TiO_2_, respectively, as well as the effects of doping. Anionic doping shifts the VB and cationic doping shifts the reduction potential. **g-h)** EPR spectra for engineered ZnO and TiO_2_, respectively. Anionic and cationic doping of ZnO, as well as anionic doping of TiO_2_, yield hydroxyl production with visible light. Cationic doping of TiO_2_ shows no radical signal – indicating reliance on the reduction potential for superoxide generation. EPR spectra for N-TiO_2_/ZnS shows clear DMPO-OOH peaks corresponding to the superoxide radical adduct

Due to several contradictory reports of ROS generation and potential “therapeutic” action of two FDA-approved materials, zinc oxide (ZnO) and titanium dioxide (TiO_2_) QDs and nanoparticles [43–49], we evaluated their redox properties and demonstrated bandgap and redox state engineering approach proposed in this review. First, unmodified or undoped ZnO and TiO_2_ nanoparticles were tested using electron paramagnetic resonance (EPR) spectroscopy technique. In order to evaluate the short-lived radical species formed from redox chemistry, we used a spin-trapping method to form more stable adducts. Both ZnO and TiO_2_ nanoparticles showed only hydroxyl radical formation upon excitation with ultraviolet light above their bandgap (Fig. 3a,b). This would be problematic for potential nanotherapy because both ultraviolet light and hydroxyl radicals are indiscriminately toxic to all cells. Careful electrochemical measurements revealed that, while the ZnO reduction potential is too low for superoxide formation, its oxidation potential is very high—leading to hydroxyl generation upon light activation **(**Fig. 3c,e**)**. Therefore, even if ZnO nanoparticles were doped with a cation (to reduce the nominal reduction potential) or anion (to reduce the oxidation potential), the visible light absorbing nanoparticles still could not form superoxide (Fig. 3e,g). This was further confirmed via electrochemical measurements by removing oxygen, where direct hole-injection into water leads to the formation of hydroxyl radical (Fig. 3c). Evaluation of TiO_2_ nanoparticles showed more promising results, however. While the oxidation potential of undoped TiO_2_ was too high, the reduction potential was suitably matched for superoxide formation (Fig. 3d, f). Therefore, anion-doped TiO_2_ nanoparticles, in principle, should form therapeutic superoxide. However, the presence of oxygen vacancies and resulting Ti^3+^ ions nominally present on this oxide material surface [50–52] catalyzes rapid Fenton chemistry to dismutate superoxide, converting it into toxic hydroxyl radicals [53]. To prevent such unwanted dismutation, we coated the surface of TiO_2_ nanoparticles with a zinc sulfide (ZnS) shell, and saw significant superoxide formation (in visible light) using anion-doped (N-doped) TiO_2_ core/ZnS shell nanoparticles (Fig. 3f, h).

### Choosing the right material(s) for QD therapeutic

While the choice of an appropriate redox-active material is important for selective therapeutic action, as shown above, the QD surface plays a key role in cellular photochemistry and biocompatibility. Many materials have appropriate redox properties to enable QD therapy, but present issues of colloidal stability or inherent cytotoxicity. A good solution to enable using such materials could be to use that material as a redox-active core, covered by a thin shell of biocompatible material [29]. Further, material cytotoxicity is often tied to a nanoparticle’s physical size, hydrodynamic radius, and surface charge (zeta potential). These factors can directly affect a nanoparticle’s affinity for surrounding biomolecules and tissue. For example, worsened zeta potential can hinder colloidal stability and potentially result in particle aggregation. This could lead to selective accumulation of QDs in some organs like kidney, spleen, and liver [17, 18], where the host cells have small pore sizes, but are quickly cleared through the rest of the organs and blood circulation. Nominally, QDs with a hydrodynamic diameter below ~ 10 nm can be cleared from the body in in vivo animal tests. After accumulating in organs, due to lack of typical metabolism as seen in small molecules, one hypothesis suggests the potential for surface material leaching to occur, causing the QDs to “shrink” in size and clear away. This release of metal elements could present unpredictable and undesired host toxicity issues. Therefore, the choice of biocompatible material, at least on the QD surface, can be critical for successful application, reducing potential toxicity concerns for host cells. Evidence in support of this hypothesis from literature can be seen in Table 1 [85–104], where different core materials (like CdSe and CdTe) when coated with other more benign/biocompatible materials, display a significant reduction in toxicity. We reviewed a number of such studies in literature with a wide range of sizes, hydrodynamic radius, and in vitro and in vivo studies. As a result, we found that even large QDs with significant retention made of/coated with less toxic elements, displayed much lower cytotoxicity. Further, materials that do not create any toxic ROS, such as hydroxyl or singlet oxygen, also displayed low cytotoxicity to the host cells. These materials could be used either as stable single-material QDs, or as coating/shell for QDs with a different core material better suited for redox perturbation.

**Table 1:**
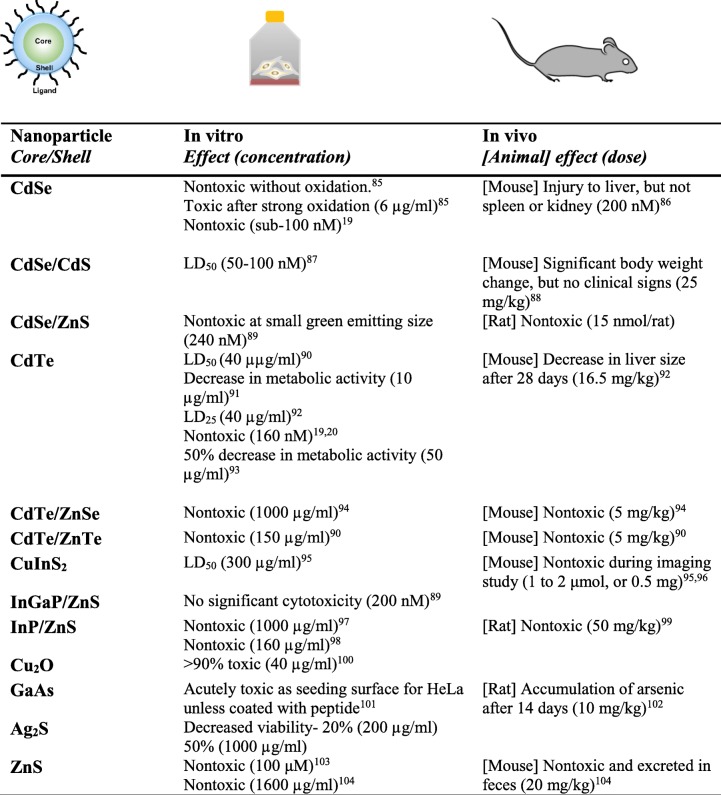
Review of in vitro and in vivo toxicity reported for relevant core/shell QDs [85–104]

### QD ligands, size, and clearance

Comparing data from identical QDs/nanoparticles with different ligands, charge, and hence resulting different hydrodynamic radius, we observed significant differences in their retention and cytotoxicity (Table 2) [18–20], [105–109]. Notably, even across materials with different toxicity, e.g. CdSe, CdTe, and Au, surface ligands clearly influence retention and cytotoxicity. At identical QD/nanoparticle core sizes, positively charged ligands (cysteamine) show indiscriminate adhesion to different negatively charged biomolecules, creating a protein “corona” that increases its hydrodynamic radius significantly. This effectively increases QD retention and resulting cytotoxicity [17, 18, 29, 54, 55]. Switching to negatively-charged ligands (mercaptopropionic acid) at same/similar core size meanwhile reduces indiscriminate biomolecule attachment and lowers/eliminates toxicity. This ligand still results in higher hydrodynamic radius and higher retention, with low/moderate toxicity. However, a similarly sized zwitterionic-ligand (cysteamine) results in low hydrodynamic radius and toxicity. These findings can be explained by the lack of formation of a protein corona and higher rates of renal clearance in in vivo animal studies. This points to a clear strategy of controlling the QD core/shell size, along with ligand and charge, so that the total hydrodynamic diameter remains below 10–15 nm. Taken together, this 3-layer design approach consists of: 1) QD made with core material with tuned reduction-oxidation potentials for selective generation of superoxide for as antimicrobial for MDR superbugs; 2) non-toxic and biocompatible shell core or shell material, resulting in high chemical stability and low material leaching and cytotoxicity; and 3) ligand design (zwitterionic) to maintain a low-hydrodynamic radius, high rates of clearance, and low toxicity, can be employed for a suitable therapeutic bottom-up design strategy for redox QD therapies.

**Table 2:**
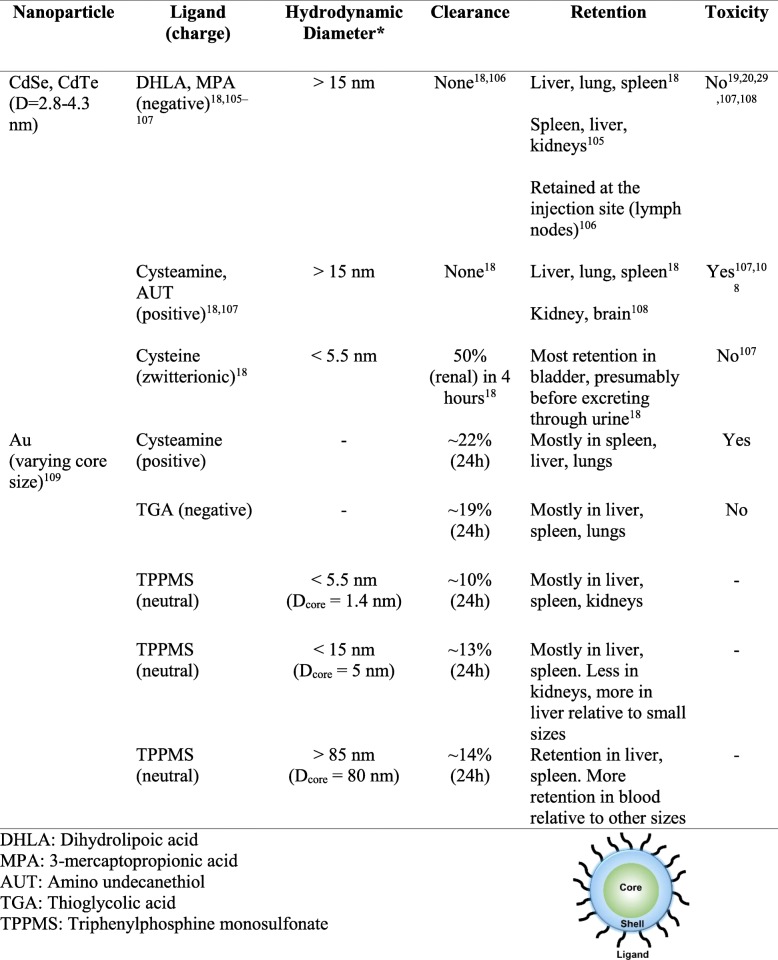
Review of common charged ligands and reported effects on biodistribution and toxicity [18–20], [105–109]

## Future outlook/approaches

### Addressing host toxicity

There are three major potential sources of toxicity for the host mammalian cells that the current and future non-traditional QD therapeutics need to address: 1) Acute material toxicity leading to loss of host cell viability or growth; 2) Oxidative stress; and 3) DNA damage and carcinogenesis. To address these concerns, the QD therapeutics first need to address acute cytotoxicity concerns in in vitro screenings, and only advance candidates that show clear differences in MIC values for the host and pathogen cells. This can be established by designing the mechanism of action after careful consideration of potential vulnerabilities in the pathogen’s cellular environment and metabolism. This, therefore, leads to a more directed and dynamic approach to counter the adaptive resistance in these MDR pathogens. Similarly, the use of specific ROS like superoxide–which can be selectively toxic to iron-sequestering pathogens–creates a clear window for differences in MIC values between host and pathogens. This therapeutic window of concentration has been successfully identified and utilized as a therapeutic in in vitro studies to target MDR pathogens, while preserving host viability and growth. Further experiments are needed to determine the transcriptomic response to superoxide therapy. More specifically, this is necessary to understand how oxidative stress from the proposed treatment affects the host. So far, the experimental evidence in literature points to non-perturbative stress response of the host to specific ROS like superoxide, and to a mechanism of action limited primarily to enzyme deactivation and indiscriminate DNA/RNA damage or genotoxicity.

**Fig. 4:**
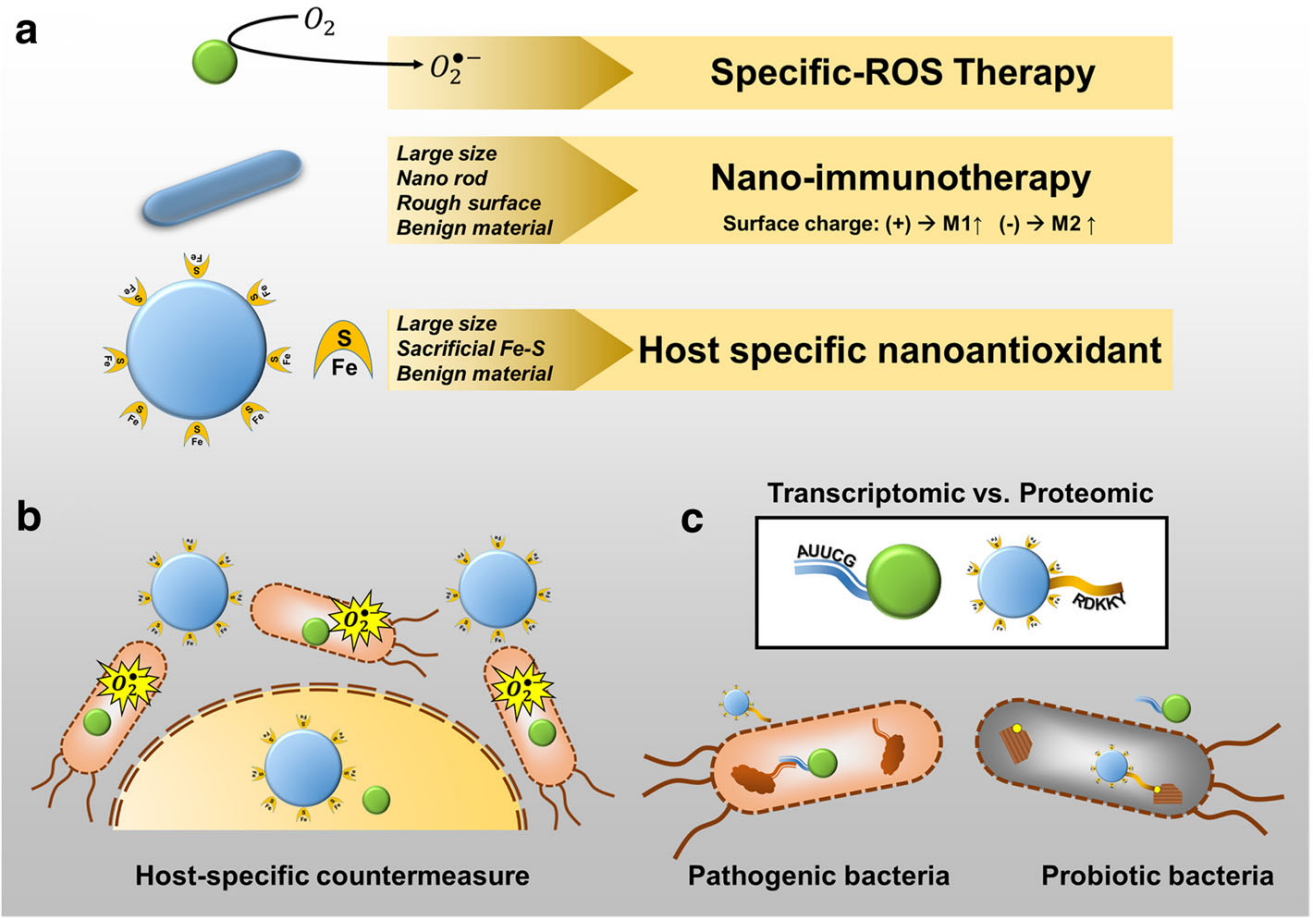
Addressing QD toxicity and future directions. **a)** Schematic and summary of three proposed types of non-traditional therapeutic, adjuvant, and countermeasure nanoparticles. QD therapeutics (top) using superoxide generation; Nano-immunotherapy (middle) using 20–50 nm benign nanorods to trigger an immune response; and a countermeasure (bottom) using large or small benign nanoparticles coordinated with Fe-S to serve as a host-specific nano-antioxidant. **b)** Depiction of host-specific protection using the larger nanoparticles coated with [Fe-S] clusters as countermeasures against the superoxide ROS stress from QD therapeutic. **c)** Depiction of probiotic-specific protection using transcriptomic/proteomic targeting with countermeasure nanoparticles

To further alleviate these concerns, our lab is developing two nanoparticle therapeutic adjuvant and “countermeasures”, made from FDA approved materials, to be supplied with the QD therapeutic: a) larger-sized adjuvant nanoparticles (< 20–50 nm) which can evoke a stronger immune response, aiding the QD nanotherapeutic [56, 57], by acting as “Nano-Immunotherapy” (Fig. 4a); and b) ~ 20–50 nm nanoparticles coated with [Fe-S] cluster [58] complexes as countermeasures, for size-selective uptake in host cells (Fig. 4b), to reduce the superoxide concentrations and ROS stress in the host and protect them against any potential toxic mechanism (Fig. 4a).

### Nano-Immunotherapeutics

are a class of new immunomodulatory materials, where their physicochemical properties: size, shape, surface charge, molecular weight, roughness, and hydrophobicity, are used to mimic normal cellular components and evade or suppress immune response (immune-evasive or immune-suppressing), or designed to inflame the host immune response for potential therapeutic effect (immune-activating materials) [56, 57]. For instnace, smaller nanoparticles have higher uptake and trafficking, allowing them to reach the lymph nodes—evoking higher levels of surface maturation markers and inflammatory cytokine secretion [59–61]. Further, asymmetrical shapes, such as nanorods, show similar trends in their immune response: Nanorods with similar radius but smaller length show higher uptake [61]. But longer nanorods induced a higher inflammatory response (IL-1α and TNF- α) because of frustrated phagocytic interactions with cells, due to their larger size [62]. Increasing the hydrophobicity of the nanomaterials surface identifies them as foreign, and potentially dangerous materials, by the immune system. This increases the gene-expression of pro-inflammatory cytokines [63]. Coating the QD or other nanomaterial surfaces with hydrophilic molecules reduces the surface protein adsorption and decreases interaction with immune cells, thereby reducing immunomodulatory response. Studies on the effect of the surface charge have shown confounding effects with other dominant physiochemical properties. Gold nanorods with positive surface charge (amine-terminated ligands) exhibit expression of anti-inflammatory surface antigens and negatively-charged (carboxylic acid-terminated ligands) surfaces induced expression of pro-inflammatory genes [64]. However, other studies have concluded that negatively-charged amino acid residues can sometimes prevent uptake of long fibrillized peptide materials by antigen-presenting cells, and hence prevent presentation of epitope peptides–thereby inhibiting immune function [65]. Overall, zwitterionic ligands or surface charges prevent accumulation/adsorption of biomolecules like proteins (biofouling), thereby evading foreign-body response [56, 57, 66]. These elements of immunomodulation were used in our QD design (small size, spherical shape, small hydrophilic ligands, and zwitterionic surface charge). By reducing the potential of non-specific inflammation of the host immune system, the potential side effects of the QD therapeutic nanoparticles could be avoided.

Other aspects of immune modulation using physiochemical properties of nanoparticles depend on their molecular weight and surface roughness. The effect of surface topography at the nanoscale, along with surface chemistry was used to understand the innate immune response. While surface acidity has a larger role in immunomodulation, surface roughness directly is correlated with enhanced matrix metalloproteinase-9 production by primary neutrophils, and a decrease in the pro-inflammatory cytokine secretion from primary macrophages [67]. This immunomodulation via surface roughness could be attributed to a reduction in inflammation and increased healing on encountering rough surfaces.

Based on the design rules summarized here, the adjuvant Nano-Immunotherapeutic will: 1) be larger-size nanoparticles than QD therapeutics (< 20–50 nm), but small enough that they easily transport to reach lymph nodes [68, 69] and initiates/upregulates the innate immune response of the body [59], to aid the QD therapeutic and fight pathogens; 2) be shaped as short nanorods, rather than spherical nanoparticles, for preferential uptake and stronger immunomodulation [64]; 3) have induced surface roughness and hydrophobicity [63, 70, 71]; and 4) have a designed surface charge to tune the inflammatory response (Fig. 4a) [64, 72, 73]. These nano-immunotherapeutic nanoparticles could, reversibly, also be used to downregulate the immune response and inflammation, in case the QD nanotherapeutic has any adverse/side-effects due to retention of over-activity. Further, the size-selected uptake of nanoparticles acting as counter-measures for the host cells would be designed to counter any ROS stress, deactivation of superoxide in the host, and reduce any potential for genotoxicity from the QD therapeutic, using a coating of [Fe-S] clusters on these nanoparticles (Fig. 4a,b) [58]. Such coatings can be easily created using a hydrophobic-hydrophilic surface interaction, and will be used with a small subset of FDA approved materials, like ZnO, TiO_2_, or silica nanoparticles.

### Improving selective uptake in different cell-types (host and pathogen)

One future approach to improving QD therapeutics is targeting selective uptake between host and pathogen (Fig. 4b), as well as between different pathogens (e.g. pathogenic vs. probiotics, Fig. 4c). Size can be an important factor in tuning uptake between host and pathogens [74, 75]. By selecting for the appropriate size, a QD therapy can selectively generate therapeutic superoxide and induce pathogen-killing, while protecting the host cells using nano-countermeasures. For selectivity between different types of bacteria, such as pathogenic strains and gut microbiota, target specificity must be considered. Reaching such targets would require the identification of the genomic, transcriptomic, or proteomic factors that separate the distinct strains. QD therapeutics can be easily coated with peptides or DNA/RNA molecules with appropriate target sequences (Fig. 4c) [76–81]. Using this methodology, similar sized pathogen-targeted QDs can be selectively uptaken by the pathogens as a QD therapeutic, while similarly sized countermeasures can be selectively transported into the probiotic bacteria, further protecting them from adverse effects of the QD therapeutic. This approach can boost the efficacy of QD therapeutics while reducing potential side-effects. Importantly, the window of QD therapeutic flux between host and pathogens can be further expanded to provide more immediate and effective relief to patients.

## Conclusions

In conclusion, this review summarizes the potential, existing, state-of-the-art, and future outlook for an emerging class of radical QD therapeutics. Here, we specifically sought to show several aspects of QD design, geared towards treating MDR superbug infections. By tailoring the stimuli-triggered photochemistry, inherent materials, and chosen mechanism of action, a bottom-up rational design strategy was outlined for the QD therapeutic. This approach begins with a mechanism of redox action that targets a specific vulnerability in the pathogen compared to the host cells. Achieving such biological specificity is important to preserve the healthy host cells and offer the safest possible treatment. Selecting a redox mechanism is then followed by careful material selection and 3-layered design to optimize safety and efficacy. The proposed approach will be bolstered by further work to develop a nanoparticle adjuvant, such as nano-immunotherapeutics, and nano-countermeasures for host and probiotic cells. While the work presented here shows a design approach to radical therapy for countering adaptive resistance in bacteria, the same approach can be easily extended to a range of different diseases (e.g. cancer), as well as to precision medicine. For precise treatment of diseases at the scale of molecular biology, healthy and diseased cells can be distinguished from each other, and QD interactions can be tailored to exploit those differences. Using this emerging Quantum Biology approach being developed in our group and by other researchers, a new rational design strategy can be achieved for therapies that are dynamic or adaptive, and can be quickly tailored at the atomic and molecular level. The semiconductor QDs discussed here can be leveraged to rationally design effective treatments, using the governing principles described in this review. Progress in this area could stimulate the development of a new class of smart therapies, reduce the time required for regulatory approval by using small tweaks in atomic and molecular arrangement of an approved QD therapeutic, and enable researchers to deploy their inventions to address a rapidly emerging class of adaptive or dynamic diseases.

## Abbreviations

Ag_2_S: Silver sulfide
AUT: Amino undecanethiol
CdS: Cadmium sulfide
CdSe: Cadmium selenide
CdTe: Cadmium telluride
CuInS_2_: Copper indium sulfide
Cu_2_O: Copper oxide
DHLA: Dihyrolipoic acid
DNA: Deoxyribonucleic acid
EPR: Electron paramagnetic resonance
FDA: Food and Drug Administration
Fe-S: Iron-sulfur
GaAs: Gallium arsenide
InGaP: Indium gallium phosphide
InP: Indium phosphide
MDR: Multidrug-resistant
MIC: Minimum inhibitory concentration
MPA: 3-mercaptopropionic acid
NHE: Normal hydrogen electrode
QD: Quantum dot
RNA: Ribonucleic acid
RNS: Reactive nitrogen species
ROS: Reactive oxygen species
TGA: Thioglycolic acid
TiO_2_: Titanium dioxide
TPPMS: Triphenylphosphine monosulfonate
ZnO: Zinc oxide
ZnS: Zinc sulfide
ZnSe: Zinc selenide
ZnTe: Zinc telluride
